# Chimeric Antigen Receptor T-Cell Therapy in Glioblastoma: Current and Future

**DOI:** 10.3389/fimmu.2020.594271

**Published:** 2020-11-03

**Authors:** Long Li, Xiqun Zhu, Yu Qian, Xiangling Yuan, Yi Ding, Desheng Hu, Xin He, Yuan Wu

**Affiliations:** ^1^ Department of Oncology, Tongji Hospital, Huazhong University of Science and Technology, Wuhan, China; ^2^ Department of Surgical Oncology, Hubei Cancer Hospital, Tongji Medical College, Huazhong University of Science and Technology, Wuhan, China; ^3^ Department of Medical Oncology, Hubei Cancer Hospital, Tongji Medical College, Huazhong University of Science and Technology, Wuhan, China; ^4^ Department of Radiation Oncology, Hubei Cancer Hospital, Tongji Medical College, Huazhong University of Science and Technology, Wuhan, China; ^5^ Institute of Human Virology, Zhongshan School of Medicine, Sun Yat-sen University, Guangzhou, China

**Keywords:** glioblastoma, brain tumor, immunotherapy, chimeric antigen receptor T cell therapy, CAR T

## Abstract

Glioblastoma (GBM) is a highly aggressive glioma with an extremely poor prognosis after conventional treatment. Recent advances in immunotherapy offer hope for these patients with incurable GBM. Our present review aimed to provide an overview of immunotherapy for GBM, especially chimeric antigen receptor T-cell (CAR T) therapy. CAR T-cell immunotherapy, which involves the engineering of T cells to kill tumors by targeting cell surface-specific antigens, has been successful in eliminating B-cell leukemia by targeting CD19. IL-13Rα2, EGFRvIII, and HER2-targeted CAR T cells have shown significant clinical efficacy and safety in phase 1 or 2 clinical trials conducted in patients with GBM; these findings support the need for further studies to examine if this therapy can ultimately benefit this patient group. However, local physical barriers, high tumor heterogeneity, and antigen escape make the use of CAR T therapy, as a treatment for GBM, challenging. The potential directions for improving the efficacy of CAR T in GBM are to combine the existing traditional therapies and the construction of multi-target CAR T cells.

## Introduction

Glioblastoma (GBM) is a highly aggressive, malignant, and undifferentiated glioma with a global incidence of 10/100,000, and frequently occurs in individuals aged between 55 and 60 years (1, 2). It is the most common type of astrocytoma with poor prognosis (3). After aggressive treatment, the median survival time is only 14–15 months post diagnosis, the 5-year survival rate is approximately 10%, and the final mortality rate is close to 100% (4). The etiology of GBM is poorly understood. To date, exposure to high doses of ionizing radiation is the only established risk factor (5). Cell phones, electromagnetic fields, occupational exposures, and formaldehyde have not been found to be associated with GBM (6). GBMs can be divided into primary GBMs (~90%) and secondary GBMs according to clinical and histological characteristics. Primary GBMs are without histologic or clinical evidence of a less malignant precursor change, whose majority develop quickly in elderly patients. Secondary GBMs progress from anaplastic astrocytoma or low-grade diffuse astrocytoma. With a less degree of necrosis, they manifest in much younger patients (7). GBMs can also be classified into isocitrate dehydrogenase (IDH) wild type, which is generally equivalent to the primary GBMs, and IDH mutant type, which is mainly secondary glioblastoma based on the 2016 World Health Organization classification for tumors of the central nervous system (8). Maximum surgical resection in combination with radiotherapy and chemotherapy (temozolomide) has become the standard therapy for newly diagnosed GBM (9, 10). However, GBM recurrence is inevitable after a median survival time of 32–36 weeks. Once the disease recurs, it becomes resistant to drug treatment and is essentially incurable (11).

Due to the poor prognosis of patients treated with conventional therapies for GBM, attention has been shifted to other emerging treatments, such as immunotherapy (12). The immune system can detect and destroy tumor cells through the process of immunosurveillance. However, some tumor cells escape immunosurveillance and gradually develop into tumor lesions. The purpose of tumor immunotherapy is to overcome the immune resistance of tumor cells in order to treat the tumor. Immunotherapy includes vaccines, oncolytic virus therapies, checkpoint blockade, and adoptive T cells (13). Tumor immunotherapy has rapidly evolved in recent years and has shown promising results in a variety of tumors such as lung cancer (14), kidney cancer (15), and melanoma (16).

The most common strategy for direct recruitment of T cells is adoptive lymphocyte transfer. Autologous T cells that target tumor cells *in vitro* are trained, amplified, and activated, and then transferred to the patient’s body. These genetically engineered T cells are specific to targeted cells and can strengthen tumor immunity. Adoptive T cells include tumor invasive lymphocytes, cytokine-induced killer cells, TCR engineered T cells, and chimeric antigen receptor T-cell (CAR T) therapies (17, 18). In fact, different immune therapies are often used in combination with other treatments rather than used alone for better clinical results. Among them, CAR T-cell therapies have achieved tremendous developments. Thus, we reviewed the current studies on CAR T-cell therapy for GBM, discussing the obstacles and future directions in this promising area of therapy.

## Overviews of CAR T-Cell Therapies in GBM

**Figure 1:**
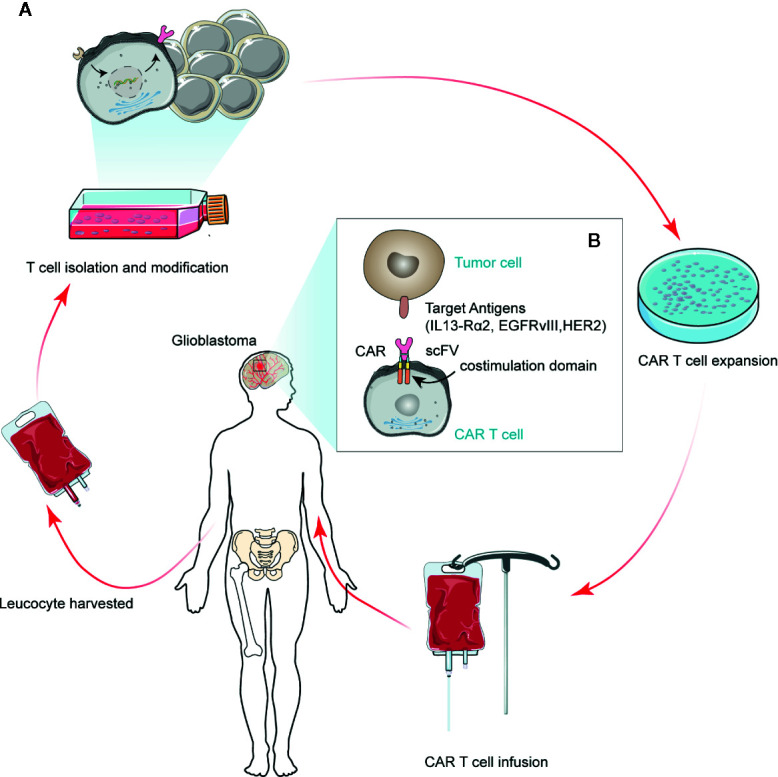
Schematic depicting regulatory CAR T therapy in GBM. A modified CAR T cell can recognize tumor cell surface antigens in an MHC-independent manner, thus inducing tumor cell death. Currently, the antigens available for clinical trials for GBM tumor cells are IL13-Rα2, HER2, and EGFRvIII. The scFv represents a single variable region of antibody expression in T cells.

CAR T cells are autologous or allogeneic modified T cells, which are collected from patients’ peripheral blood, amplified *in vitro*, and remolded genetically to express CAR molecules on the cellular membrane *via* viral vectors or electroporation. Their extracellular domains could recognize tumor-specific antigens, while their intracellular domains contain T-cell activation signals. These modified T cells are then administered to the patient’s body, where they could lyse cells that carry the relevant tumor antigens (19). The general flow of CAR T treatment in GBM is shown in **Figure 1**. Physiological antigens can recognize the TCR-CD3 complex in the extracellular region, which has six independent gene products: TCR α, β chains, and CD3 g, δ, ϵ, and ζ chains. The TCR α and β chains could bind to the HLA-peptide complex. The CD3 γ, δ, ϵ, and ζ chains can activate T cells (20). The intracellular signal domain of activated T cells usually contains a signaling domain, which could be recognized as the first-generation CAR without other signal domains. The addition of a co-stimulatory signal domain, usually CD28 or 41BB, produces a second-generation CAR. The third-generation of CAR is generated by the combination of several different co-stimulus proteins and multiple co-stimulus domains (21). This would induce the production of T cells and lead to the killing of cancer cells by cytotoxic cells (22, 23).

By 2020, the FDA has approved two CAR T-cell therapies for CD19+ B cell malignancies, named Yescarta and Kymriah (24). In a phase 2 study on patients with relapsed or refractory acute B-cell lymphoblastic leukemia, up to 81% of the patients experienced remission 3 months after CAR T-cell therapy. After 6 months, the survival rate was 73%, and the event-free rate was 90%. Moreover, after 12 months, the survival rate was 50%, and the event-free rate was 76% (25). Another multicenter phase 1–2 study, participated by 22 institutions, reached a similar conclusion (26).

In addition to successful clinical practice in above malignant hematological diseases, many clinical trials of CAR T therapy have also been carried out in other solid malignancies, including GBM (27), colorectal (28), pancreatic (29), renal cell (30), ovarian (31), and breast cancers (32). Although CAR T therapy has not yet entered clinical practice for solid tumors, it has given hope to patients with other cancer types who have few treatment options. The following chapters will focus on the progress of CAR T therapy in GBM.

## Clinical Application of CAR T-Cell Therapies in GBM

To date, due to the lack of tumor-specific antigens expressed in GBM, the application of CAR T cells in GBM is still limited (33). However, with the emergence of the second- and third-generation CAR, it is possible to overcome the low heterogeneity of GBM tumors and achieve a better clinical effect. In order to cover the published trial results in this area, we searched PubMed and ClinicalTrials.gov (https://clinicaltrials.gov/ct2/home) for GBM trials conducted until June 2020. We found 18 clinical trials regarding CAR T-cell therapy for GBM, including trials on different CAR T-cell targets and different therapy combination strategies such as combined chemotherapy or immune checkpoint blockade. However, only three studies related to CAR T-cell targets published the clinical responses. **Table 1** shows that interleukin-13 receptor alpha 2 (IL13-Rα2) (37), human epidermal growth factor receptor 2 (HER2) (36), and epidermal growth factor receptor variant III (EGFRvIII) (35) have been clinically verified as effective and safe targets of CAR T-cell therapy for GBM.

**Table 1:** Published clinical trials of CAR T therapy in GBM.

Study	Target	Results
Goff et al. (34)	EGFRvIII	No clinically meaningful effect was evaluated in 18 patients.
O’Rourke et al. (35)	EGFRvIII	Nine patients had a stable condition for 28 days, while the rest showed disease progression at day 28.
Ahmed et al. (36)	HER2	One patient showed partial response for 9 months, seven had a stable condition for 8 weeks to 29 months, and eight experienced disease progression.
Brown et al. (37)	IL13-Rα2	One patient achieved complete response for 7.5 months.
Brown et al. (38)	IL13-Rα2	IL13-Rα2-specific CAR T cells could be used in the treatment of GBM.

### IL13-Rα2

IL13-Rα2 is a monomer of IL13 with a high affinity receptor. It is overexpressed in almost all tumors related to the glomerular basement membrane, but not in healthy tissues. IL13-Rα2 is rarely expressed in normal brain cells but is highly expressed in GBMs. This specificity made it an ideal target for CAR T-cell therapy in GBM. With the increase in malignancy, the expression of IL13-Rα2 also increased. IL13-Rα2 is also considered as a prognostic indicator (39, 40).

In the immune system, IL13 is usually expressed in the sharing receptors with its homologue IL4 in several normal tissues, modulating the immune responses. A previous study showed that almost all GBMs highly express IL13 receptors. In contrast to other tissues, the IL13 receptors on GBMs are independent of IL4, leading to the discovery of IL13-Rα2 (40).

In 2004, researchers described a novel method for targeting GBM multiforme with IL13-Rα2-specific CAR T cells by their genetic alteration to express a membrane-tethered IL13 cytokine chimeric T-cell antigen receptor (also known as zetakine). The adoptive transfer of IL13-zetakine (+) CD8(+) CTL clones led to the regression of verified human GBM orthotopic xenografts *in vivo* (41). In 2015 and 2016, Brown et al. reported that several patients with recurrent and refractory GBM received CAR T cells targeting IL13Rα2. As this therapy was well tolerated, the patients’ brain inflammation was temporarily managed. After treatment, the overall expression of IL13-Rα2 in some patients decreased, while the tumor necrotic volume increased at the site of IL13-zetakine(+) T-cell administration (37, 38). Subsequently, the structure of CAR T cells was optimized to achieve a better clinical effect. In 2018, Brown described the optimization of IL13-Rα2-targeted CAR T cells. They designed a 4-1BB (CD137) co-stimulatory CAR (IL13BBzeta) and constructed a manufacturing platform using enhanced central memory T cells. This study revealed that IL13BBzeta-CAR T cells increased the T-cell persistence and anti-tumor activity. Moreover, compared with intravenous administration, the CAR T cells’ local intracranial delivery elicited better anti-tumor efficacy. However, intraventricular infusions exhibited more benefits than intracranial tumor infusions in a multifocal disease model (42).

### HER2

HER2 is a transmembrane tyrosine kinase receptor expressed in various normal tissues. This protein participates in the development and progression of several tumors, such as breast cancer, ovarian cancer, gastric cancer, osteosarcoma, and medulloblastoma (43, 44).

In animal models, CAR T cells targeting HER2 presented better anti-tumor activity and survival rate (45). Being a validated immunotherapy target for GBM, HER2 is expressed in nearly 80% GBM patients. Generated from GBM patients, HER2-specific T cells could target their CD133+ stem cell compartment and autologous HER2-positive GBMs (46).

Another clinical study consisting of 10 consecutive GBM patients revealed that HER2-specific T cells could stimulate T-cell proliferation and secretion of IFN-gamma and IL-2 in HER2+ autologous GBM cells. Derived from primary HER2+ GBMs, these HER2-specific T cells could killed CD133+ and CD133− cells, whereas HER2-negative tumors were not killed (46). Another study included 17 patients with progressive HER2+ GBM. Without prior lymphodepletion, they received one or more autologous HER2-CAR VST mixtures. Being well tolerated, infusions did not show any dose-limiting toxic effects. Among these 16 evaluable patients, 1 showed a partial response for over 9 months, 7 had a stable condition for 8 weeks to 29 months, 8 had disease progression after T-cell infusion during the 24–29 months of follow-up, and 3 with stable conditions survived without any signs of disease progression (36). Moreover, combined with other targets, HER2 is often applied in the study of second- or third-generation CAR T-cell therapy, which would be explained in detail in the following combined therapies.

### EGFRvIII

EGFRvIII is expressed in the absence of wild-type EGFR proteins, which produces constitutionally active receptor (ligand independent) and two distantly combined epitopes from the extracellular domains. EGFRvIII was initially discovered in a primary human GBM, which was expressed in nearly 30% of GBM samples. In addition to poor prognosis, EGFRvIII could enhance proliferation, angiogenesis, and invasion of glioma cells (47). Highly tumor-heterogeneous EGFRvIII can induce phenotypic transformation. It is overexpressed in GBM cells, but not in normal cells. Considering these findings, EGFRvIII is an effective target for CAR T cell therapy in GBM (48).

In 2014, Miao observed that in areas with invasive tumor, EGFRvIII-CAR T cells were overexpressed, which suppressed the tumor growth and improved the survival time of mice (49). A study of 10 patients with recurrent GBM reported that the manufacturing and infusion of EGFRvIII-CAR T cells are safe and profitable, without evidence of cytokine release syndrome or off-tumor toxicity (35). However, other studies have shown that EGFRvIII-CAR T cells have a limited effect on GBM. In 2019, another clinical study involving 18 GBM patients who were treated with anti-EGFRvIII CAR T cells, showed a median progression-free survival time of 1.3 months with a single outlier of 12.5 months. Although cell dose would influence the persistence of CAR cells, objective responses were rarely observed. In this phase I pilot trial, the application of anti-EGFRvIII CAR T cells did not show clinically meaningful effects in GBM patients (34).

Subsequently, more trials focused on the modification of anti-EGFRvIII CAR T cells, such as BiTE-EGFR CAR T cells, PDIA3 mutant EGFRvIII CAR T cells, and EGFR806-CAR T cells. They improved the efficacy and safety of CAR T cells as a treatment for GBM (50, 51).

### Novel Targets

Considering the profound tumor heterogeneity of GBM, scientists have been exploring new effective targets for GBM. Cluster of differentiation 70 (CD70) is overexpressed in glioma cells, but not in peripheral and normal brain tissues, and is associated with immune-related cell infiltration (52). This finding suggests that CD70 may be a potential new CAR T therapeutic target for GBM; however, further studies are still needed. Glioma-associated antigen ephrin type A receptor 2 (EphA2) is highly expressed primarily in the GBM cells of the brain. EphA2 has successfully exhibited an anti-tumor activity as a target for CAR T therapy in a glioma xenograft model; however, data on the duration of remission are limited (53). Chondroitin sulphate proteoglycan 4 is not only highly expressed in GBM specimens but it also has limited heterogeneity, which can be induced by TNFα. The use of this antigen has been shown to be effective against glioma cells in an *in vitro* CAR T therapy study (54). B7-H3, also known as CD206, is highly expressed in most malignant tumors, including high-grade brain tumors and sarcomas, but is rarely expressed in normal human tissues (55, 56). Although the role of B7-H3 in immune regulation remains unclear, there is no doubt that its overexpression is associated with tumor metastasis, invasion, and malignancy (57). Therefore, it is an attracting target for cancer immunotherapy. B7-H3-specific monoclonal antibodies, MGA271 (58) and 8H9 (59), have shown anti-tumor effects in preclinical mouse model studies and are well tolerated in phase I clinical trials (60). One CAR T therapy targeting B7-H3 has indicated good anti-tumor activity on GBM at both cellular and mouse levels (61). Chlorotoxin, a natural 36-amino acid peptide, has the potential bind to GBM while showing minimal cross-reactivity with normal cells in the brain (62). This provides an opportunity to expand target antigens for GBM CAR T therapy. Chlorotoxin-CAR T therapy presents a strong anti-tumor effect in patient-derived GBM cell lines and mouse xenograft models without significant toxicity to normal cells (63). In addition, the novel antigenic targets currently reported are summarized in **Table 2**. However, none of these targets have achieved the results of previous clinical trials.

**Table 2:** Published novel tumor antigen targets in CAR T therapy for GBM.

Study	Target	Conclusions
Jin et al. (64)	CXCR1-or CXCR2	CXCR1 or CXCR2 modified CAR T cells were capable of tumor regression in the GBM preclinical model.
Tang et al. (61)	B7-H3	B7-H3 is overexpressed in GBM patients and can be a therapeutic target.
Yang et al. (65)	NKG2D-BBz	NKG2D CAR-T cells targeted glioblastoma cells and cancer stem cells in an NKG2D-dependent manner.
Wallstabe et al. (66)	alphavbeta3	Alphavbeta3 can enhance CAR reactivity.
Yi et al. (53)	EphA2	EphA2-CAR T cells therapy has been shown to be effective in a preclinical model.
Pellegatta et al. (54)	CSPG4	The expression level of CSPG4 in GBM was high and the heterogeneity was not obvious.
Ge et al. (67)	CD70	CD70 is associated with tumor progression.
Zhu et al. (68)	CD57	CD57 was significantly upregulated in activated human T cells.
Wang et al. (63)	Chlorotoxin	Chlorotoxin-CAR T therapy mediated potent anti-tumor activity in the orthotopic xenograft GBM models.

## Obstacles and Improve Strategies in CAR T Therapy for GBM

Although several studies have been conducted and advancements have been made on CAR T for GBM, the actual clinical effect of CAR T in GBM is not promising, which is mainly caused by physical barriers, antigen escape, and tumor heterogeneity. Although GBM has been shown to be complex in immunotherapy, several strategies have shed some light on the increased efficiency of CAR T in this disease.

### Blood-Brain Barrier

**Figure 2:**
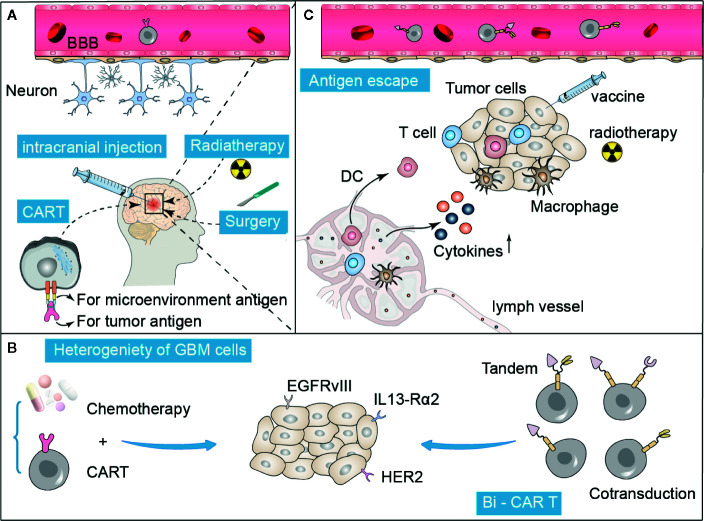
Summary of difficulties and possible improvement strategies in CAR T treatment for GBM. **(A)** The specific anatomy of the blood-brain barrier (BBB) prevents the entry of many drugs, including CAR T cells, which can be overcome to some extent by intracranial direct injection, radiation, or surgery. **(B, C)** Tumor heterogeneity and antigen escape are the two major reasons that limit the efficacy of CAR T therapy. These obstacles can be overcome by administering a combination of traditional treatments, such as chemotherapy, radiation, and adjuvant vaccines. In addition, the construction of multi-target specific CAR T cells is also a promising approach.

Historically, the brain has been recognized as an immune-privileged region. The lack of traditional lymphatic vessels and well-known antigen-presenting cells further underpinned this theory (69). Thus, GBM is an immunologically quiet tumor. In addition, the blood-brain barrier (BBB) prevents the entry of almost all large molecules and 98% of small molecules to the central nervous system, limiting the effective delivery of drugs to the tumor site (70) (**Figure 2A**).

Immune checkpoints are molecules on the surface of activated T cells that act as “brakes” to prevent the occurrence of inflammatory responses due to an immunodeficiency. The classic checkpoint of CTLA-4 or PD-1 could result in the inactivation or even death of T cells. Blockage or antagonization of these signals can persistently activate the production of T cells (71). Checkpoint blockade showed promising clinical efficacy in many tumors; however, GBM is often resistant (72). Compared with other tumors that are responsive to immunotherapy, GBM has a notoriously low mutational burden, resulting in the less production of tumor-infiltrating T cells (73, 74). In addition, repeated immune activation in the intracranial space would promote clinical hazards, including cytokine release syndrome and autoimmune encephalitis (75).

### Heterogeneity of GBM and Antigen Escape

GBM is also a remarkably heterogeneous tumor that facilitates immune escape, which may be the largest obstacle (13, 76, 77). According to the gene expression analysis, GBM can be divided into four subtypes: typical, neuroural, proneural, and mesocytic (78). Even within the same tumor specimen, GBM showed significant heterogeneity. Sottoriva et al. found that there were different subtypes of tumor fragments in different spaces of GBM (79). The presence of heterogeneity made it difficult to implement the CAR T therapy and decreased the effectiveness of immunotherapy (80, 81). CD19 CAR trials showed that a major cause of resistance to treatment is the loss of target antigen. Moreover, the loss of antigen will likely block the effect of CAR therapy in solid tumors. A potential approach to overcome this obstacle is combinational targeting of multiple GBM antigens to enhance the tumor killing activity and reduce the antigen escape.

It is difficult to identify a single universal antigen for GBM because of the highly complex tumor heterogeneity in both different GBM patients and among different GBM subtypes (82). However, multiple antigen-targeted CARs with multiple specificity, including CAR T hybrid cell populations expressing two antigens in tandem or multiple antigens to overcome tumor heterogeneity (**Figure 2B**), have shown encouraging anti-tumor efficacy and safety in preliminary studies (83). T cells co-expressing HER2 and IL13-Rα2 CARs could effectively target and kill tumor cells that express either HER2 or IL13-Rα2, and showed particularly enhanced anti-tumor activity and antigen-dependent downstream signals compared with a single targeting strategy (45). Trivalent CAR T cells, targeting HER2, IL13-Rα2, and EphA2, exhibited excellent anti-tumor activity *in vitro* in the GBM model (84). Although combination therapies have shown promise in addressing tumor heterogeneity, further optimization is needed in terms of the number and combination of target antigens.

GBM contains self-renewing and multipotent subpopulation of cells, defined as cancer stem cell (CSC) that contributed to tumorigenesis, recurrence, and high therapeutic resistance (85). GBM appears to originate not from a single cell type but from a variety of seed cells, suggesting heterogeneity in CSC themselves (86). The discovery of CSC and its role in the pathogenesis of GBM suggest that it may be a new therapeutic target. IL13-Rα2 specific CAR therapy has been shown to kill both GBM cells and CSC in animal models (87). A preclinical study of oncolytic virus therapy based on neural stem cell delivery has shown great promise in GBM (88). This suggests that therapies targeting stem cells may be beneficial in overcoming tumor heterogeneity and antigen escape. CD133 positive CSCs are present in a variety of solid tumors, including GBM (89). In a phase I clinical study of CD133 targeted CAR T therapy for 23 advanced digestive system malignancies, 3 patients achieved partial remission and 14 had stable disease, showing good anti-tumor efficacy and controlled toxicity (90). Compared to CD133 monoclonal antibody therapy and dual antigen T cell engager antibody therapy, CD133 specific CAR T had enhanced activity in patient-derived models of GBM without acute systemic toxicity (91). In addition, others such as CD15, integrin α6, CD44, and L1CAM have also been suggested as potential markers of CSCs (92). Given that most of these markers are also present in normal stem cells, targeting these markers need to be studied with caution, because it may result in a potential overlap between the CSC and the stem cells of normal cells. A more specific CSC surface marker may be one of the best treatment options in the future.

### Combination With Traditional Therapy Approaches

Immune cells, including T cells, are severely restricted from entering the brain because of the BBB (93). How to efficiently transfer the modified CAR T cells to tumor lesions in the brain still needs to be explored further. Systematic and regional delivery methods have been successfully used to enhance the trafficking of CAR T cells to the tumor sites (94). Radiotherapy and surgery can damage the BBB to some extent, which provides a promising option to be combined with CAR T therapy. Immune checkpoint inhibitors combined with stereotactic radiotherapy have shown superior efficacy in preclinical glioma models (95). A study showed that direct intratumoral injection improved the anti-CAIX CAR-T potency by restricting its off-target effects (96) (**Figure 2A**). However, there may be a lower risk of off-target toxicity. Further studies are needed to describe and compare the T-cell persistence and overall therapeutic effect associated with regional and systemic delivery of CAR T-cell therapy. Pre-treatment with chemotherapy can reduce the production of regular T cells and activate the anti-tumor response. In relapsed/refractory chronic lymphocytic leukemia, CD-19-targeted CAR T therapy following conditional chemotherapy increases its efficacy (97). Bevacizumab, a vascular endothelial growth factor inhibitor, increases the lymphocyte infiltration and inhibits the occurrence of immunosuppression caused by VEGF. A previous study showed that GD2 CAR-T cell therapy combined with bevacizumab can enhance its anticancer efficacy in a preclinical model of neuroblastoma (98). In gliomas, temozolomide chemotherapy is usually associated with the occurrence of lymphotoxicity. Sequential CAR T therapy after a dose-intensified regimen of temozolomide chemotherapy has been shown to improve CAR engraftment and anticancer activity in rodent models (99). In addition, CAR T cells, with the aid of anticancer vaccines, significantly extended the survival in a GBM mouse model, and no significant side effects were observed (100). Conventional therapies, such as chemotherapy and radiation, as well as vaccines, may help CAR T therapy overcome the problems of tumor heterogeneity and antigen escape in patients with GBM (**Figures 2B, C**).

## Conclusion

Immunotherapy has revolutionized the overall treatment strategy for many solid tumors and is also expected to improve the response of GBM patients to treatment. In the treatment of GBM, CAR T-cell therapies, especially the second- and third-generation CAR T-cell therapies, have achieved promising preclinical efficacy to prolong the survival time of patients. However, due to the location and particularity of GBM, no phase III clinical trial results have been published. Hence, further studies are needed to modify CAR T cells and their targets to improve their clinical efficacy.

## Author Contributions

LL and XZ wrote the paper. LL drew the figures. YW, XH, XY, and DH designed the framework and content of the article. YQ and YD searched the literatures. YW and XH reversed the article. All authors contributed to the article and approved the submitted version.

## Funding

YD was partly supported by the National Natural Science Foundation of China (Grant no. 11704108).

## Conflict of Interest

The authors declare that the research was conducted in the absence of any commercial or financial relationships that could be construed as a potential conflict of interest.
